# Bulk and single cell RNA sequencing data reveal lactylation-related gene signatures for prognosis and immunity in cervical cancer

**DOI:** 10.1590/1414-431X2026e15368

**Published:** 2026-08-21

**Authors:** Jing Song, Yutian Zhao, Chunlin Dong, Xiaowei Qi, Qu Guo, Yongju Zhuang, Chunqing Yu, Ruofan Dong

**Affiliations:** 1 Department of Gynecology, Affiliated Hospital of Jiangnan University, Wuxi, Jiangsu Province, China; 2 Department of Radiotherapy, Affiliated Hospital of Jiangnan University, Wuxi, Jiangsu Province, China; 3 Department of Pathology, Affiliated Hospital of Jiangnan University, Wuxi, Jiangsu Province, China

**Keywords:** Cervical cancer, Lactylation-related genes, Prognosis, Immune landscape, Personalized therapy

## Abstract

Previous studies indicate that lactylation, a post-translational modification, may play an important role in the progression of cervical cancer (CC). However, the comprehensive roles of lactylation in influencing the tumor microenvironment, immune landscape, and prognosis of CC have yet to be fully elucidated. The bulk RNA-seq and single-cell RNA-seq datasets of CC patients were downloaded from the Cancer Genome Atlas and Gene Expression Omnibus databases, respectively. A total of 630 genes associated with lactylation activity were identified using AUCell algorithm, differential expression, and correlation analyses. Subsequently, a prognostic risk model comprising 17 genes was constructed through univariate Cox and LASSO analyses, which accurately predicted the prognosis of CC patients and was validated in an independent dataset. The nomogram, including risk scores and N staging, outperformed other clinical parameters. The high-risk group was positively related to the glycolysis pathway. Furthermore, bioinformatics analyses further indicated that the high-risk group was associated with a poorer prognosis, pro-tumorigenic pathways, and immunosuppression, and was sensitive to ulixertinib, dasatinib, nutlin-3a, and trametinib. Mendelian randomization analysis suggested that *ITGA5* may be causally associated with an increased risk of CC, with caution in causal inference. Additionally, the expressions of several risk genes were validated using real-time qPCR on tissue samples from six CC patients. In conclusion, the lactylation-related gene risk model could accurately and independently predict the prognosis of CC patients, providing insights into therapeutic strategies for CC patients. These bioinformatics-based findings are exploratory and warrant further validation in preclinical and clinical settings.

## Introduction

Cervical cancer (CC) is a major global health challenge, with high morbidity and mortality rates, making it one of the most common malignancies among women (1). Human papillomavirus (HPV) is acknowledged as the primary risk factor for CC. Increasing CC screening and vaccination efforts is crucial for enhancing patient survival rates (2). However, the global disease burden remains substantial (3). The prognosis for patients with advanced-stage CC is notably poor, and outcomes worsen further with tumor invasion, metastasis, or recurrence (4). Emerging therapeutic strategies, such as targeted therapies and immunotherapies, are currently the focus of intense research in managing CC (5). Due to patient heterogeneity, some individuals with CC may exhibit resistance to immunotherapy, targeted therapy, or chemotherapy (6). Therefore, identifying novel therapeutic targets is essential to improve outcomes for CC patients.

Tumor cells primarily rely on aerobic glycolysis instead of oxidative phosphorylation, converting glucose into a large amount of lactate, a metabolic process known as the Warburg effect (7). The production of lactate meets the metabolic demands of tumor proliferation and creates a hypoxic, nutrient-deficient, and acidic tumor microenvironment (TME) (8). Furthermore, lactate is associated with key carcinogenic processes, including invasion, metastasis, angiogenesis, and immune evasion (9). Lactylation, a post-translational modification, involves the covalent attachment of lactate to the lysine residues on proteins, playing a significant regulatory role in immune cells and cancer metabolism (10). In HeLa cells, 3,633 lysine lactylation (Kla) sites have been identified, involving 1,637 different proteins (11). Additionally, histone lactylation may influence the prognosis of CC by modulating the tumor immune microenvironment (12). However, the association between lactylation-related gene signatures and prognosis in patients with CC has not been fully elucidated.

In this study, we comprehensively characterized lactylation-related genes (LRGs) using bulk RNA sequencing (RNA-seq) and single-cell RNA sequencing (scRNA-seq) of CC patients from public genomic databases. Subsequently, we developed a prognostic risk model and analyzed its implications in clinical prognosis, immune infiltration, somatic mutations, and drug sensitivity, providing new perspectives for the diagnosis and treatment of CC.

## Material and Methods

### Bulk RNA-seq data download and processing

The RNA-seq data (HTSeq-FPKM), relevant survival information, and clinicopathological characterization data of The Cancer Genome Atlas (TCGA) cervical cancer (CESC) were downloaded from the University of California, Santa Cruz (UCSC) Xena database (https://xena.ucsc.edu/). The RNA-seq data (gtex_RSEM_gene_fpkm) of normal cervical tissues were acquired from Genotype-Tissue Expression (GTEx) (https://xenabrowser.net/datapages/). The data of GTEx were merged with TCGA-CESC data and converted to log_2_(fpkm+1) to alleviate distributional skewness. Subsequently, the 291 primary tumor tissue samples and 13 normal cervical tissue samples were obtained for further analysis. Standardization was performed using the normalizeBetweenArrays function in the limma package to reduce technical variation across samples. Additionally, the RNA-seq data of CGCI-HTMCP (HIV^+^ Tumor Molecular Characterization Project) -CC were downloaded from the Genomic Data Commons (GDC, https://portal.gdc.cancer.gov/). A total of 118 primary tumor samples with available survival information were obtained for external validation.

### scRNA-seq data download and processing

The scRNA-seq dataset GSE168652, which includes tumor and normal adjacent tissues from a patient with CC, was downloaded from the Gene Expression Omnibus database (https://www.ncbi.nlm.nih.gov/geo/query/acc.cgi?acc=GSE168652). The “Read10×()” function from “Seurat” package in R was employed to process Seurat objects containing gene expression data in each sample. Quality control was performed on the samples using the following criteria: 1) ≤10% mitochondrial genes; 2) ≤5% hemoglobin genes; 3) ≤30% ribosomal genes; and 4) gene counts in the range of 200-7,000. The number of highly variable genes was set to 3,000, and data integration was performed using the SCT correction method. The cell markers and InferCNV algorithm were used for cell clustering analysis. The major cell types were identified by their markers using the “FindNeighbors” and “FindClusters” functions: lymphocytes (*CD27*, *PRF1*), macrophages (*CD163*, *FCGR2A*), fibroblasts (*COL1A2*, *APOD*), endothelial cells (*PECAM1*, *EGFL7*, *EMCN*), smooth muscle cells (*ACTG2*), and tumor/epithelial cells (*EPCAM*, *CDH1*, *CDKN2A*). Subsequently, tumor cells and normal epithelial cells were distinguished by inferring copy number variations (CNVs) using the “InferCNV” package in R. Fibroblasts served as a reference for normal copy numbers. Cells with high and low CNV were clustered by unsupervised clustering (K-means), with those exhibiting higher CNV scores classified as malignant cells.

### Identification of LRGs

A total of 332 LRGs were obtained from previous research (13) and their lactylation activity was scored by the “AUCell” package in R. All cells were divided into high and low lactylation groups based on the median area under the curve (AUC) score. Differentially expressed genes (DEGs) between the high and low lactylation groups were identified using the “PrepSCTFindMarkers” function from “Seurat” package in R with false discovery rate (FDR) <0.05 and |log_2_FC| ≥1. Correlation analysis between genes and AUC score was performed through the “psych” package in R to identify genes strongly related to lactylation activity, and the top 100 were chosen to merge with DEGs to obtain high lactylation-related genes (HLRGs), representing the most potential impact on lactylation activity.

### Functional enrichment analysis of HLRGs

Gene Set Variation Analysis (GSVA), Gene Ontology (GO), and Disease Ontology (DO) analyses were performed using “ClustProfiler”, “GSVA”, and “DOSE” packages in R to identify specific biological pathways enriched in high and low lactylation groups.

### Construction and validation of the LRG risk model

To construct a model with clinical predictive value, a univariate Cox analysis was performed on the HLRGs to screen for prognostic HLRGs (P<0.05). Subsequently, LASSO regression analysis was performed using the “glmnet” package in R. In this process, a regularization step was applied to reduce the risk of overfitting. Furthermore, the optimal regularization parameter, λ, was chosen through 10-fold cross-validation to identify the optimal number of prognostic HLRGs (as risk genes). To ensure reproducibility, a fixed random seed was set (set.seed=9). The risk score was calculated based on the risk genes profile and the regression coefficients, with the following formula: risk score = Σ[Exp(gene)×Coef(gene)], where Exp (gene) represents the expression level of risk genes and Coef (gene) represents the coefficient of risk genes in the LASSO regression analysis. Then, CC patients were randomly divided into train and test groups in a 1:1 ratio. Patients were further categorized into high- and low-risk groups based on the median risk score. Overall survival (OS) was compared between the high- and low-risk score groups by Kaplan-Meier (KM) analysis using the “survival” and “survminer” packages, and P<0.05 was considered statistically significant. Receiver operating characteristic (ROC) curves were generated by the “timeROC” package to validate the accuracy of the risk model in predicting patient survival.

### Comprehensive analysis of risk scores with different clinicopathologic characteristics

We investigated the relationship between risk scores and clinical characteristics. The prognostic prediction effects of risk scores in patients across different clinical characteristics (age, grade, M stage, and T stage) were assessed using the KM method and the log-rank test (P<0.05).

### Development and validation of clinicopathologic nomogram

Univariate and multivariate Cox analyses were performed to assess the prognostic independence of risk scores and clinical variables. A nomogram was developed using “rms” packages, including risk scores and clinical variables as prognostic factors. The scores for prognostic factors were added to predict the 1-, 3-, and 5-year survival. The accuracy of the nomogram was assessed using calibration curves. The clinical utility of the nomogram was assessed by decision curve analysis (DCA) using the “ggDCA” package in R.

### Functional enrichment analysis between high- and low-risk groups

GSVA and gene set enrichment analysis (GSEA) were used to explore the functions of genes in the high- and low-risk groups and identify the difference in the lactylation-related pathways between tumor and normal tissues. Additionally, a correlation analysis between the high- or low-risk groups and different hallmark pathways was performed to further validate GSVA results. FDR<0.05 was considered statistically significant.

### Immune function analysis

Six algorithms, “CIBERSORT”, “MCP counter”, “EPIC”, “xCell”, “TIMER”, and “QUANTIseq”, were employed using the “IOBR” package in R to assess the level of immune cell infiltration in the TME. Spearman's correlation analysis was used to calculate the relationship between risk scores and immune cell infiltration. The expression levels of several important immune checkpoint genes (ICGs) were compared between two different risk groups. The tumor immune dysfunction and exclusion (TIDE) algorithm was initially used to assess potential differences in immunotherapy response between two risk groups.

### Tumor mutation burden analysis

Somatic mutation data were downloaded from TCGA, and the mutation status of genes in the high- and low-risk groups was assessed using the “maftools” package in R. The co-mutation status of risk genes with highly mutated genes was also evaluated.

### Drug prediction and drug sensitivity analysis

A differential expression analysis was performed on risk genes between tumor and normal tissues. Drugs targeting these risk genes were predicted through the DSigDB database available on the Enrichr platform (http://amp.pharm.mssm.edu/Enrichr). The GDSC1 dataset was downloaded from the Genomics of Drug Sensitivity in Cancer (GDSC) database to obtain information on drug sensitivity. The differences in half maximal inhibitory concentrations (IC50) of different drugs were compared between the high- and low-risk groups, and their correlations with risk genes were calculated.

### Mendelian randomization analysis between risk genes and CC

Expression quantitative trait loci (eQTL) summary statistics for risk gene expression in blood, as well as genome-wide association study (GWAS) CC summary statistics were obtained from the IEU Open GWAS Project (https://gwas.mrcieu.ac.uk/), which provides harmonized and quality-controlled summary-level data across a wide range of traits and diseases. The GWAS dataset for CC (GCST90018817) included 909 cases and 238,249 controls of European ancestry. Mendelian randomization (MR) analysis was conducted using the TwoSampleMR R package as previously described (14). Briefly, single nucleotide polymorphisms (SNPs) strongly associated with gene expression (exposure) were selected using a significance threshold of P<5×10^-6^. To ensure independence among instrumental variables (IVs), linkage disequilibrium (LD) clumping was performed with a threshold of “r^2^<0.001, 10,000 kb”. Only SNPs with an F-statistic >10 were retained to avoid weak instrument bias. The inverse-variance weighted (IVW) method was used as the primary MR approach. Heterogeneity across instruments was assessed using Cochran's Q test, and sensitivity was evaluated using a leave-one-out analysis.

### Real-time qPCR

Tumor and matched adjacent normal tissue samples were collected from six patients with CC for validation of selected risk genes. These CC patients were recruited from the Affiliated Hospital of Jiangnan University. The inclusion criteria were as follows: 1) newly diagnosed CC confirmed by histopathological examination; 2) no prior treatment before diagnosis, including radiotherapy, chemotherapy, immunotherapy, or molecular targeted therapy; 3) no history of other malignant tumors; 4) no concomitant autoimmune diseases; and 5) age between 18 and 70 years. The exclusion criteria were: 1) presence of other malignant tumors; 2) receipt of neoadjuvant chemotherapy, radiotherapy, or targeted therapy before surgery; 3) incomplete clinical data; and 4) any prior history of cancer. CC tissues and matched adjacent normal tissues were obtained immediately after surgical resection. The specimens were rinsed with sterile normal saline to remove blood stains, cut into appropriately sized pieces, and placed into cryovials containing RNA preservation solution. The samples were kept at 4°C overnight and subsequently stored at -80°C for long-term preservation until further experimental use. Total RNA was extracted from tissues using TRIzol reagent according to the manufacturer's instructions (Invitrogen, USA). First-strand cDNA was synthesized using the FastKing RT Kit (TIANGEN, China). Real-time qPCR was subsequently performed using SYBR Green Master Mix (TIANGEN) with gene-specific primers (BIOMED, China). Relative gene expression levels were calculated using the 2^−ΔΔCt^ method. The differences between normal and tumor groups were compared using *t*-tests. Primer sequences are listed in Supplementary Table S1.

This study was approved by the Medical Ethics Committee of the Affiliated Hospital of Jiangnan University (WXSY-YXLL-AF/SC-11/02.0). Written informed consent was obtained from each participant.

## Results

### Identification of LRGs in CC based on scRNA-seq data

**Figure 1:**
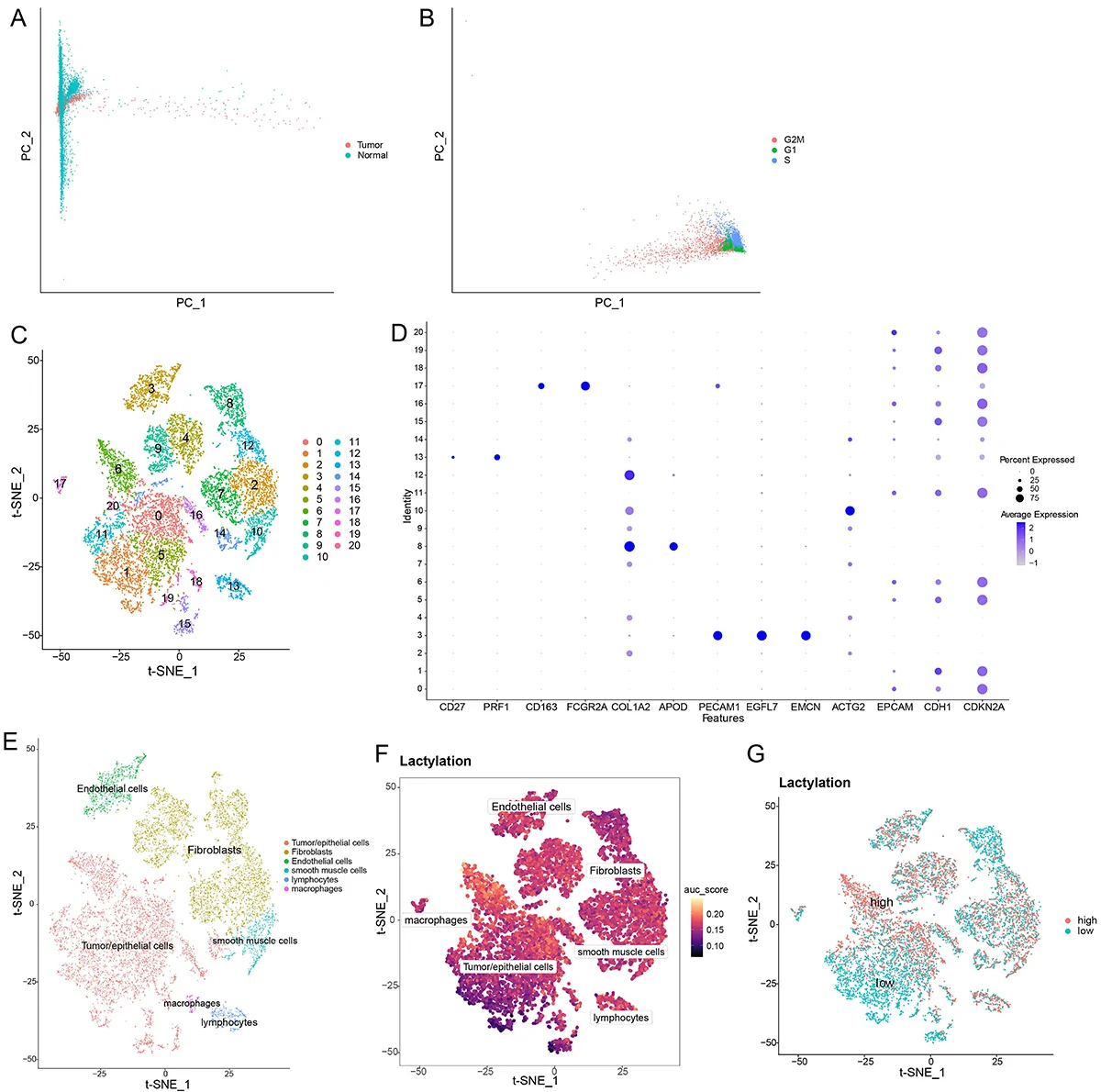
Single-cell RNA sequencing analysis. **A**, Principal component analysis (PCA) plot of samples. **B**, PCA plot of cell cycle. **C**, t-SNE plot of cell clustering. **D**, Expression of marker genes across different clusters. **E**, t-SNE plot of cell annotations. **F**, Lactylation scoring for each cell. **G**, Distribution of cells with high lactylation scores and low lactylation scores.

**Figure 2:**
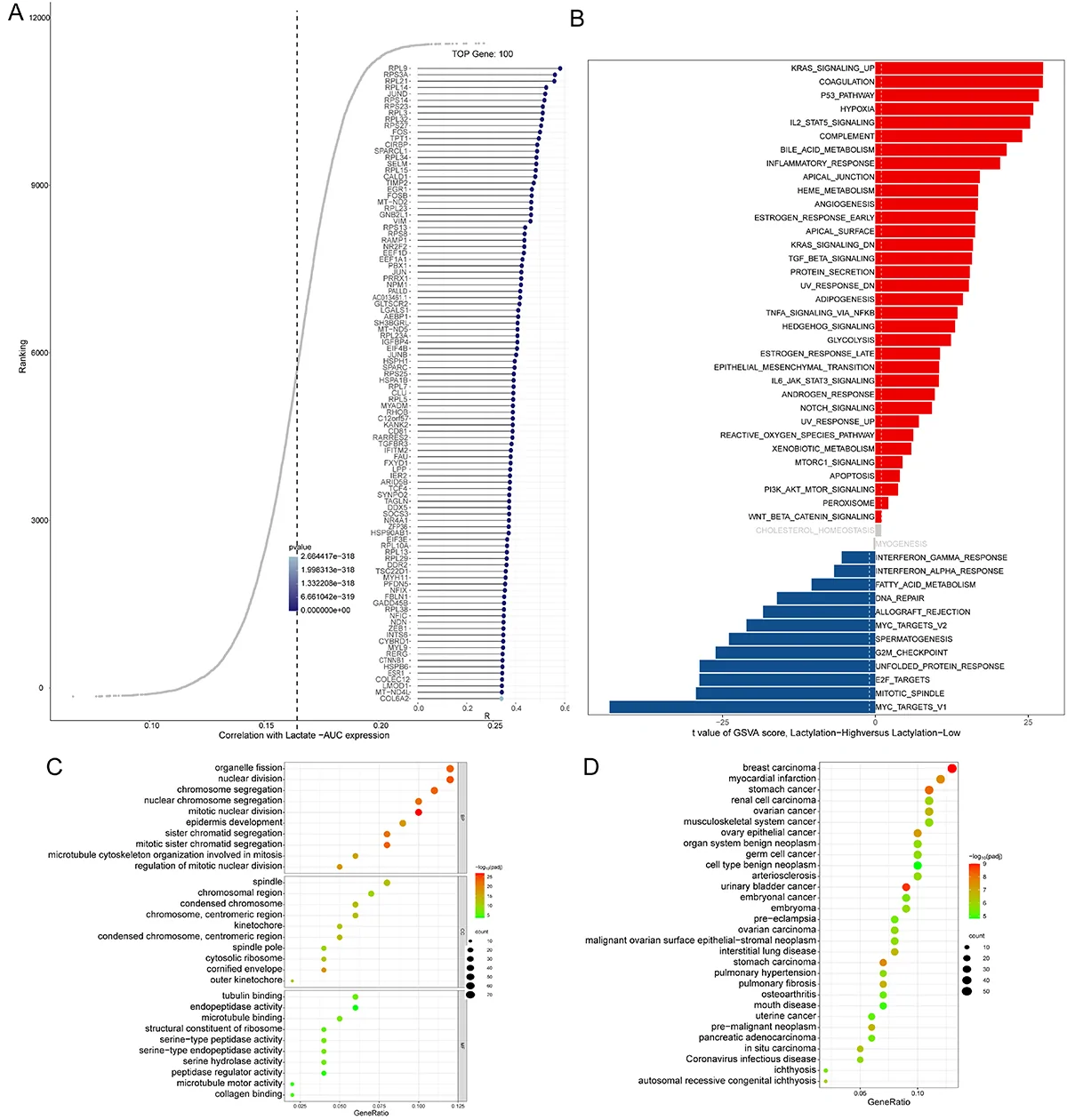
Functional enrichment analysis. **A**, Correlation analysis showing the top 100 genes most strongly associated with lactylation scores. **B**, Gene Set Variation Analysis (GSVA) enrichment. **C**, Gene Ontology (GO) enrichment (top 30). **D**, Disease Ontology (DO) enrichment (top 30).

We employed scRNA-seq data from a single CC patient for an exploratory investigation into LRGs. Principal component analysis (PCA) analysis showed that there was no significant batch effect between the two groups (Figure 1A), and the cell cycle genes clustered efficiently (Figure 1B), indicating the reliability of the data and enabling further analysis. All cells were clustered into 20 clusters using the k-nearest neighbor (KNN) clustering algorithm based on their surface markers (Figure 1C and D). After cell annotation, 6 cell types were identified, including lymphocytes, macrophages, fibroblasts, endothelial cells, smooth muscle cells, and tumor/epithelial cells (Figure 1E). Tumor/epithelial cells were then categorized into malignant and normal epithelial cells based on CNV scores (Supplementary Figure S1A-C). Next, all cells were classified into high and low lactylation groups based on the median AUC score. t-SNE visualized the distribution of LRGs across different cell subsets (Figure 1F and G, Supplementary Figure S1D and E). Subsequently, we identified 530 DEGs between the high and low lactylation groups. Correlation analysis revealed the top 100 genes most strongly associated with lactylation activity (Figure 2A).These genes were merged with DEGs to obtain 630 HLRGs that may be related to lactylation activity. GSVA, GO, and DO analyses of HLRGs revealed that lactylation may play important roles in immune pathways (“IL2_STAT5_SIGNALING” and “TGF_BETA_SIGNALING”) and pro-tumor pathways (“PI3K_AKT_mTOR” and “HYPOXIA”) (Figure 2B-D).

### Identification of risk genes and their cellular localization

**Figure 3:**
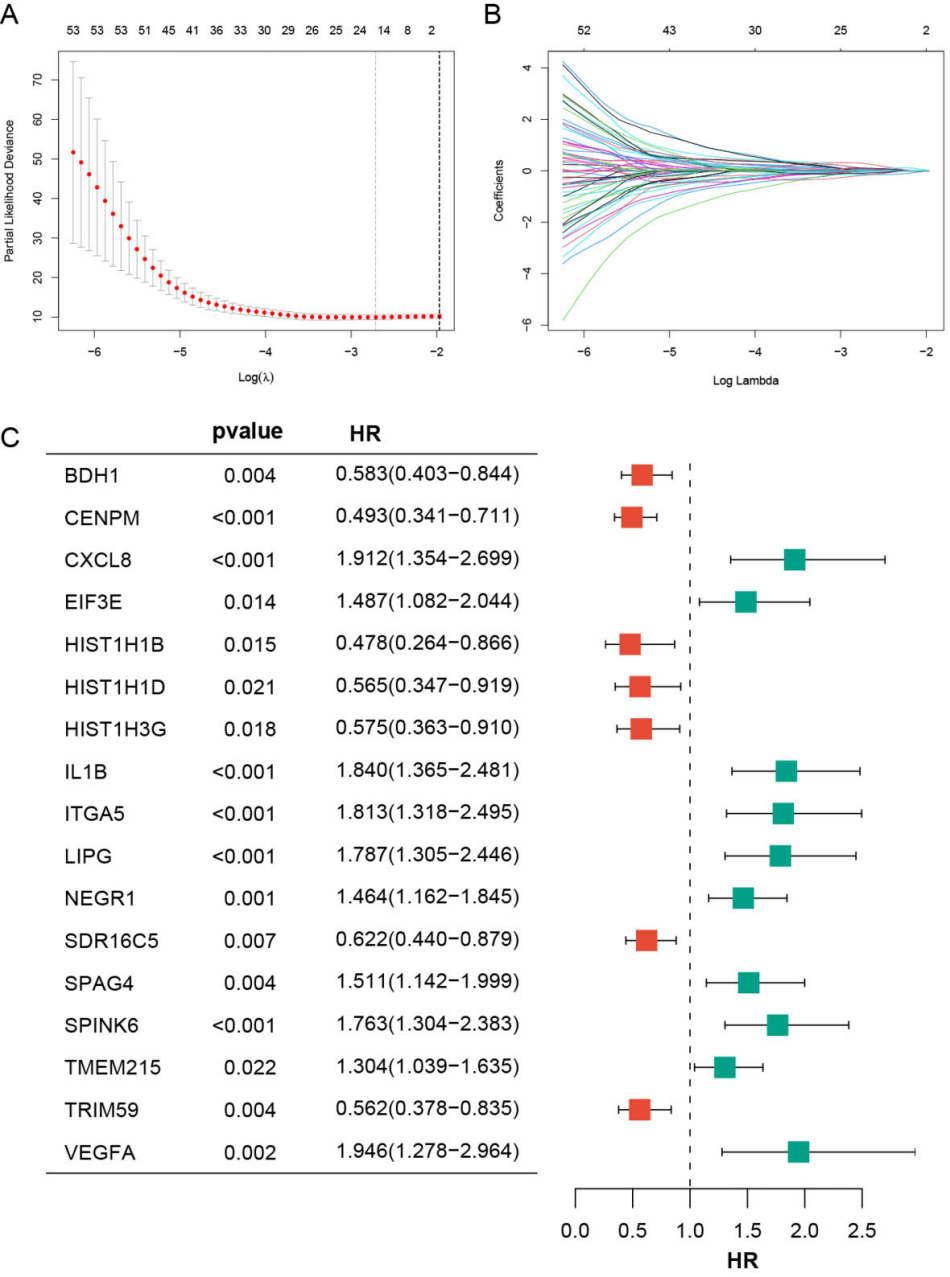
Identification of risk genes and their expression patterns analysis. **A** and **B**, Ten-fold cross-validation for variable selection in the Least Absolute Shrinkage and Selection Operator algorithm. **C**, Forest plot showing the hazard ratios (HRs) of the 17 risk genes identified through LASSO-Cox regression analysis for CC.

Univariate Cox analysis was performed on 630 HLRGs and the results revealed 63 HLRGs that were associated with prognosis, including 16 favorable factors and 47 risk factors. Subsequently, LASSO regression analysis was performed to identify the 17 risk genes, including *BDH1*, *CENPM*, *CXCL8*, *EIF3E*, *HIST1H1B*, *HIST1H1D*, *HIST1H3G*, *IL1B*, *ITGA5*, *LIPG*, *NEGR1*, *SDR16C5*, *SPAG4*, *SPINK6*, *TMEM215*, *TRIM59*, and *VEGFA* (Figure 3A-C). We used scRNA-seq data to investigate the distribution of risk genes in different cell types. The results showed *CXCL8* and *IL1B* were mainly expressed in macrophages, *ITGA5* was mainly expressed in endothelial cells, *SDR16C5* in malignant cells, and *VEGFA* in malignant cells, normal epithelial cells, fibroblasts, smooth muscle cells, and macrophages, while *EIF3E* was expressed in all cells (Supplementary Figure S2).

### LRG risk model accurately predicted the prognosis for CC

A risk model consisting of these 17 risk genes was constructed. LASSO algorithm was used to calculate the risk score for each tumor sample as shown in the following formula: risk score = (-0.0327×BDH1 expression)+(-0.1317×CENPM expression)+(0.2811×CXCL8 expression)+(0.0836×EIF3E expression)+(−0.0040×HIST1H1B expression)+(-0.1484×HIST1H1D expression)+(-0.0069×HIST1H3G expression)+(0.0458×IL1B expression)+(0.0557×ITGA5 expression)+(0.0887×LIPG expression)+(0.0432×NEGR1 expression)+(-0.1758×SDR16C5 expression)+(0.1026×SPAG4 expression)+(0.0776×SPINK6 expression)+(0.0177×TMEM215 expression)+(-0.0874×TRIM59 expression)+(0.0009×VEGFA expression). Two hundred and ninety-one CC patients were randomized in a 1:1 ratio into a train cohort (n=145) and a test cohort (n=146). Patients were categorized into high- and low-risk groups based on the median risk score.

**Figure 4:**
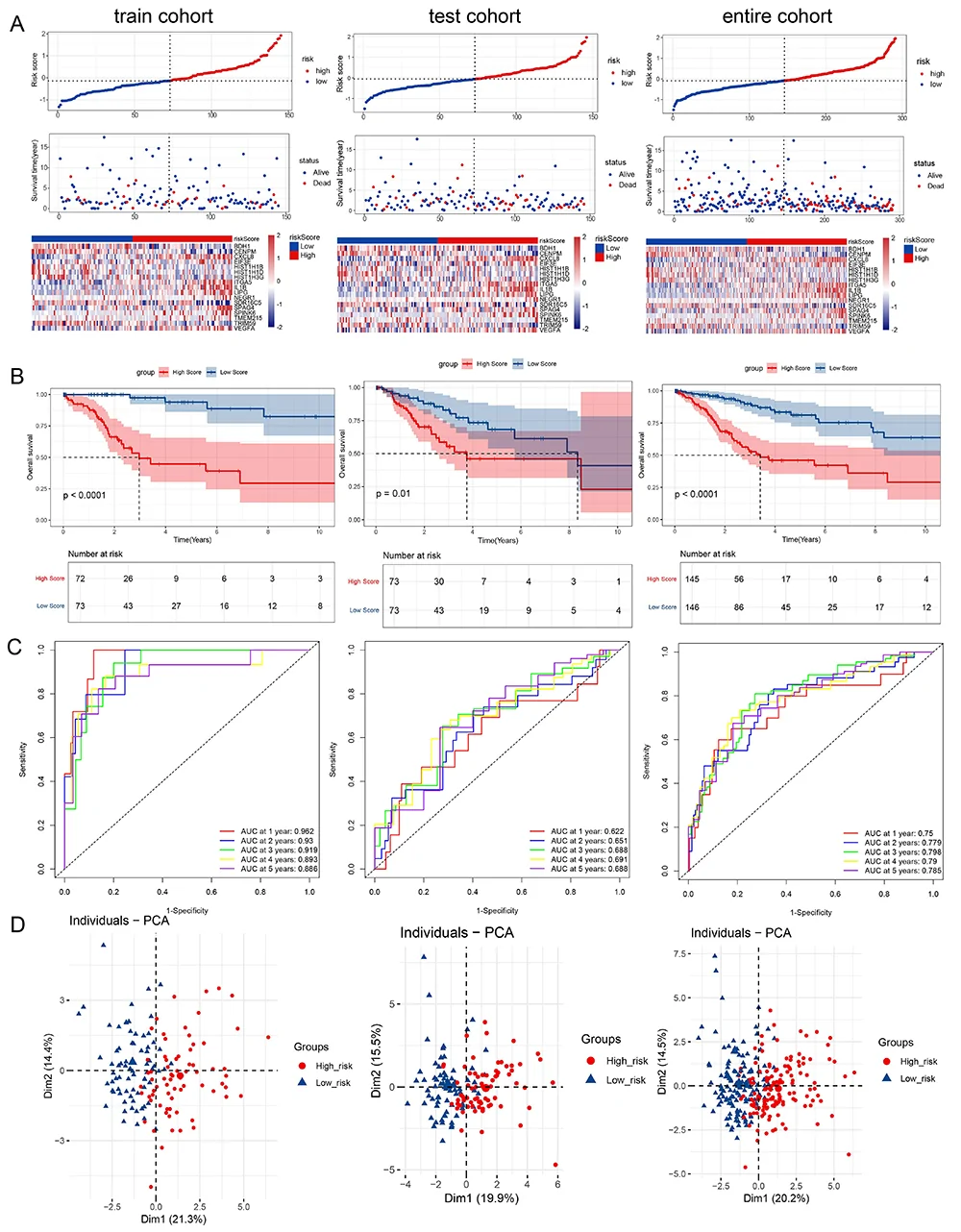
Lactylation-related genes (LRG) risk model for cervical cancer (CC) prognosis in the train cohort (left), test cohort (center), and entire cohort (right). **A**, Scatter plot of risk scores and survival status, and heatmap of the expression of 17 risk genes. **B**, Kaplan-Meier survival curves for overall survival of patients with high- and low-risk scores. **C**, ROC curves for 1-, 2-, 3-, 4-, and 5-year predictions. **D**, Principal component analysis (PCA) plot of patient samples in high- and low-risk groups.

We explored the relationship between risk score and survival status, and the results revealed a positive correlation between higher risk scores and poorer survival in each cohort, with consistent differential expression of the 17 risk genes between the high- and low-risk groups (Figure 4A). KM survival analyses showed that patients in the low-risk group had significantly longer survival than those in the high-risk group in each cohort (Figure 4B). The ROC curves indicated that the AUC for 1-, 2-, 3-, 4-, and 5-year survival was near or above 0.9 in the train cohort, and the AUC was above 0.6 in the test cohort, demonstrating the predictive ability of the risk model for survival in patients with CC (Figure 4C). PCA plots clearly showed the two different distribution patterns of samples between the high- and low-risk groups in each cohort (Figure 4D). We also obtained RNA-Seq data from the CGCI-HTMCP-CC cohort for external validation (Supplementary Figure S3). The validation results were generally consistent with those from the TCGA-CESC entire cohort.

### Comprehensive analysis of risk scores with different clinicopathologic characteristics

**Figure 5:**
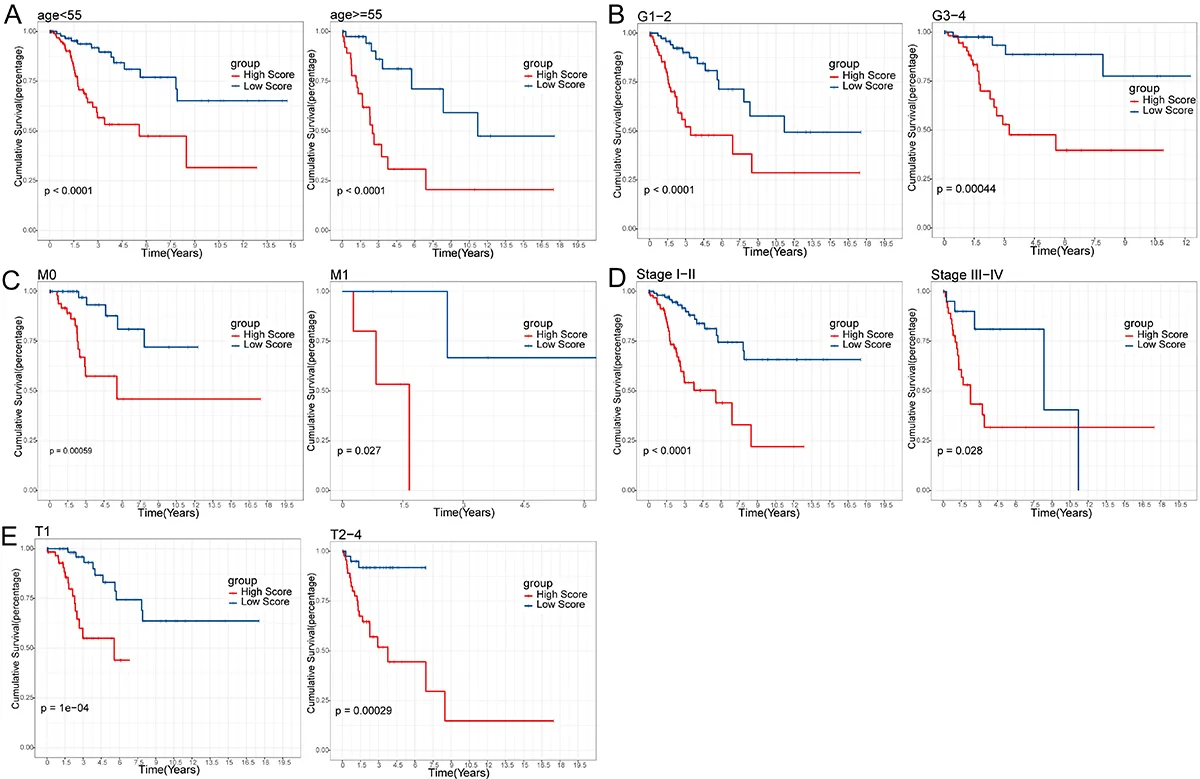
Association analysis of risk score with clinicopathological features. **A**, Age. **B**, Grade staging. **C**, M staging. **D**, Stage. **E**, T staging.

We further assessed the prognostic value of the risk score in different clinicopathologic characteristics and found that patients in the low-risk group had a better survival outcome than those in the high-risk group, both in different ages (Figure 5A), grade staging (Figure 5B), M staging (Figure 5C), N staging (Figure 5D), and T staging (Figure 5E) subgroups. These results suggested that the risk model could assess the prognosis of patients with CC in different clinical subgroups.

### Development of nomogram and independent prognostic analysis

**Figure 6:**
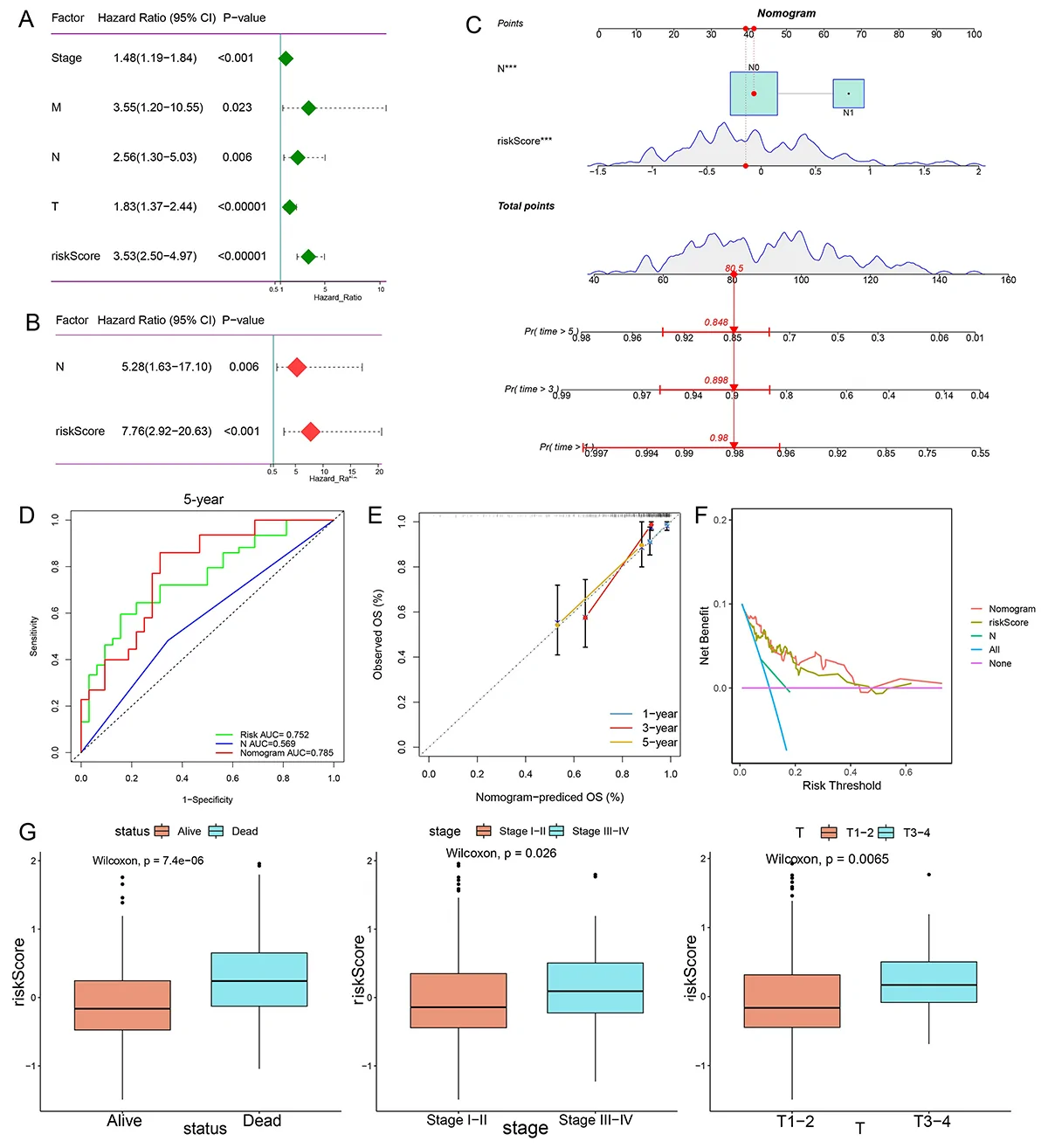
Lactylation-related genes (LRG) risk score-related nomograms and clinical characteristics for precision prediction. **A**, Univariate Cox regression analysis of risk score and clinical characteristics. **B**, Multivariate Cox regression analysis of risk score and clinical characteristics. **C**, Nomograms that include risk score and N staging predicted 1-, 3-, and 5-year survival. **D**, ROC curves comparing the risk score, N staging, and nomogram for predicting 5-year survival. **E**, Calibration curves for predicting 1-, 3-, and 5-year survival. **F**, Decision curve analysis. **G**, Differences in risk scores between different clinical subgroups (survival, Stage, T staging). Data are reported as median and interquartile range. ***P<0.001. Mann-Whitney test.

To determine whether risk score independently predicted the prognosis of patients with CC, we performed univariate and multivariate Cox analyses. Univariate Cox analysis showed that risk score, Stage, M staging, N staging, and T staging were considered as risk factors for CC (Figure 6A). Multivariate Cox analysis showed that risk score and N staging could be used as independent prognostic factors for CC (Figure 6B). Subsequently, a nomogram containing risk score and N staging was developed for predicting patient survival at 1, 3, and 5 years (Figure 6C). Clinical ROC curves showed that the AUCs of the risk score, N stage, and nomogram for predicting 5-year overall survival were 0.752, 0.569, and 0.785, respectively (Figure 6D). The AUCs of the risk score, N stage, and nomogram for 3-year (0.864, 0.615, and 0.884) and 1-year survival (0.755, 0.611, and 0.772) were also evaluated (Supplementary Figure S4A and B). These results indicated that the risk score exhibits good prognostic accuracy in CC, while the nomogram achieved numerically higher AUC values than the isolated risk score model. The calibration curves demonstrated that the actual survival probability was highly consistent with the nomogram, suggesting potential prognostic predictive ability of the nomogram (Figure 6E). Furthermore, DCA results indicated that the nomogram was more effective than other clinical indicators in predicting the survival of CC patients (Figure 6F). The prognostic performance of the nomogram was further validated in the independent CGCI-HTMCP-CC cohort (Supplementary Figure S4C). The nomogram achieved AUCs of 0.628 and 0.764 for predicting 1-year and 2-year survival, respectively (Supplementary Figure S4D and E), which were higher than those of the isolated risk score and N stage, suggesting a consistent predictive advantage in an external dataset. The calibration curves demonstrated the potential prognostic predictive ability of the nomogram in the CGCI-HTMCP-CC cohort (Supplementary Figure S4E). Additionally, patients with advanced CC had higher risk scores (Figure 6G).

### Immune landscape and tumor mutational burden

**Figure 7:**
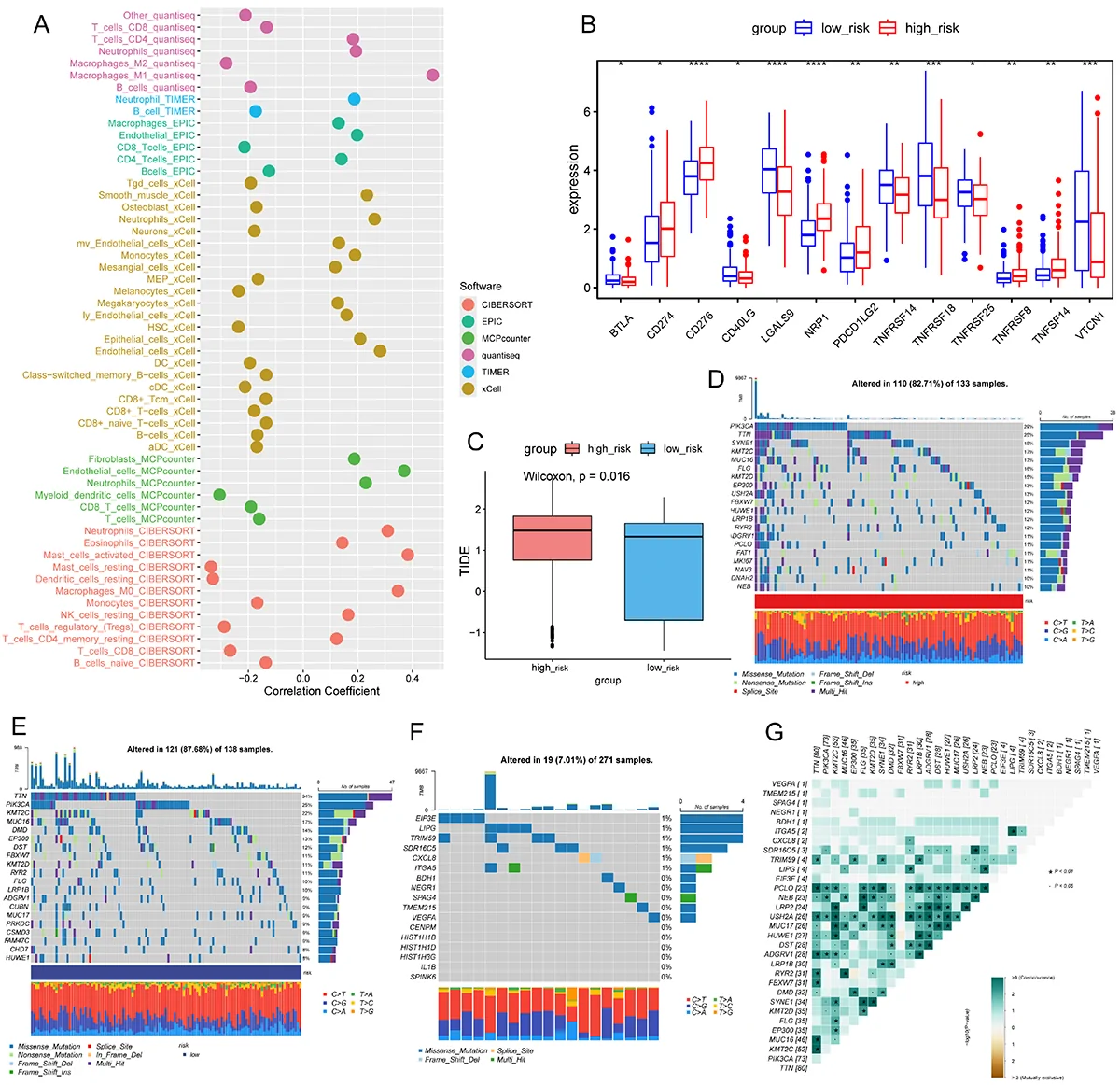
Immunity landscape and tumor mutational burden analysis. **A**, Correlation between immune cell infiltration and risk score. **B**, Differences in immune checkpoint expression levels between high- and low-risk groups. **C**, Differences in tumor immune dysfunction and exclusion (TIDE) scores between high- and low-risk groups. Data are reported as median and interquartile range. *P<0.05, **P<0.01, ***P<0.001. Mann-Whitney test. **D**, Somatic mutation waterfall plot for the high-risk group. **E**, Somatic mutation waterfall plot for the low-risk group. **F**, Somatic mutation waterfall plot for risk genes. **G**, Co-mutation status between risk genes and common mutation genes.

We assessed the level of immune cell infiltration in CC patients using six algorithms. The results showed that the LRG risk score negatively correlated with the infiltration levels of B cells and CD8^+^ T cells, and positively with those of neutrophils (Figure 7A). Additionally, significant differential expression of 14 ICGs was observed between the high- and low-risk groups (Figure 7B). Importantly, *CD274* (PD-L1) and *PDCD1LG2* (PD-L2) expressions were higher in the high-risk group. TIDE is widely used to predict patients' response to immunotherapy. TIDE scores showed that patients in the high-risk group had higher TIDE scores, suggesting that patients in the high-risk group have a higher likelihood of immune evasion and a lower likelihood of benefiting from immunotherapy (Figure 7C). Subsequently, gene mutations in the high- and low-risk groups were analyzed. The results showed that the gene mutation frequencies in the high- and low-risk groups were 82.71 and 87.58%, respectively (Figure 7D and E). Notably, *TTN* mutations were more frequent in the low-risk group compared to the high-risk group. Analysis of the mutation profiles of 17 risk genes showed no substantial mutational burden, but co-mutations with the highly mutated genes *TTN*, *PIK3CA*, *KMT2C*, and *MUC16* (Figure 7F and G).

### Functional enrichment analysis

**Figure 8:**
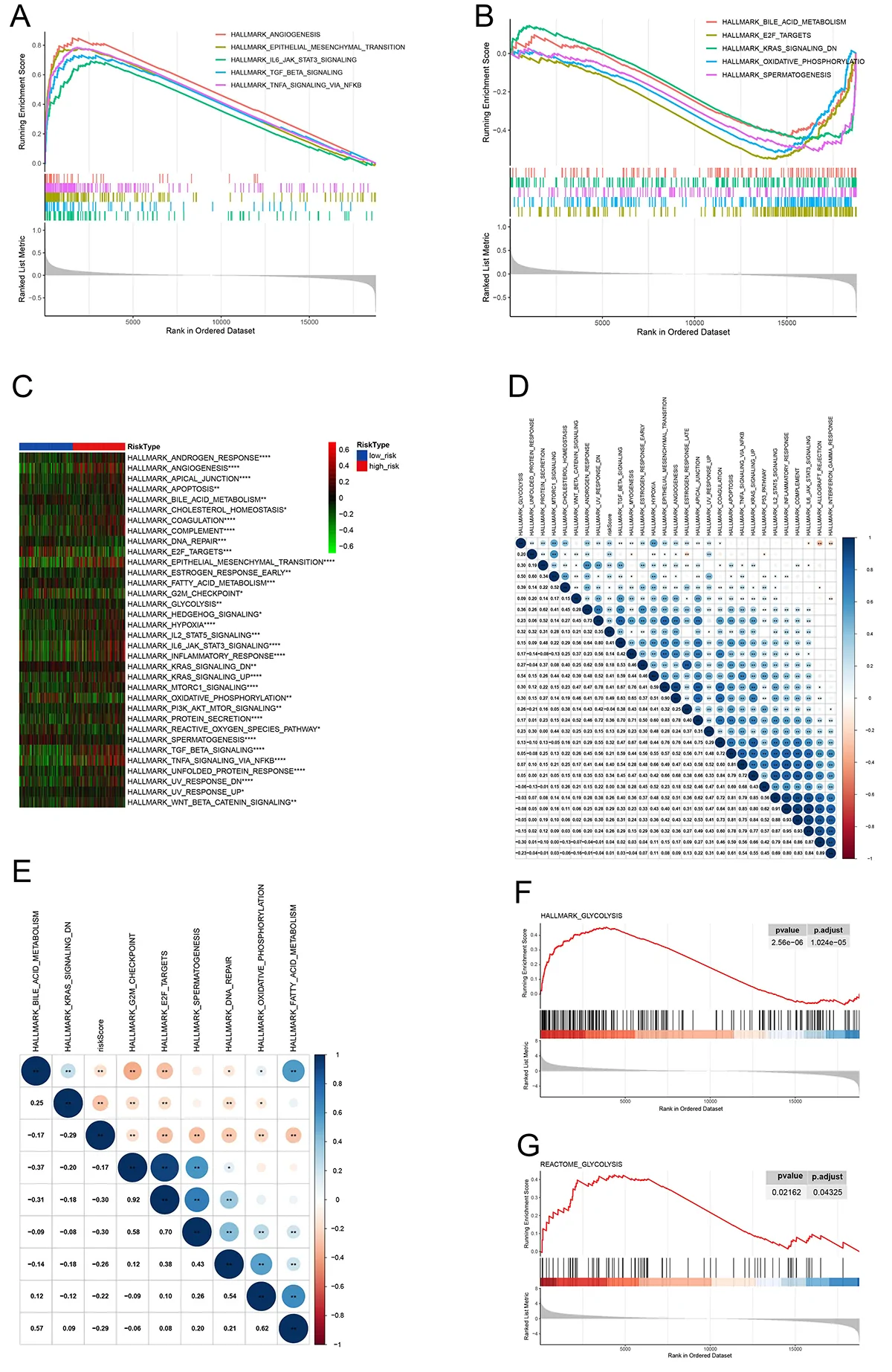
Functional enrichment analysis. **A**, Predominant functional enrichments in the high-risk group through Gene Set Enrichment Analysis (GSEA). **B**, Predominant functional enrichments in the low-risk group through GSEA. **C**, Pathway differences between high- and low-risk groups through Gene Set Variation Analysis (GSVA). **D**, Correlation between risk score and hallmark pathway activity in the high-risk group. **E**, Correlation between risk score and hallmark pathway activity in the low-risk group. **F** and **G**, Analysis of glycolysis pathway enrichment in tumor and normal tissues using Hallmark and Reactome gene sets.

Functional enrichment analysis was performed to analyze the differences in biological processes between high- and low-risk groups to reveal the reasons for the poor prognosis of the high-risk group. “MESENCHYMAL TRANSITION”, “IL6 JAK STAT3 SIGNALING”, “TGF BETA SIGNALING”, and “TNFA SIGNALING VIA NFKB” were enriched in the high-risk group. “BILE ACID METABOLISM”, “E2F TARGETS”, and “KRAS SIGNALING DN” were enriched in the low-risk group (Figure 8A and B). GSVA showed differential expression of 34 pathways (Figure 8C). Moreover, correlation analysis of signature pathway activities with risk scores further revealed that high-risk groups were associated with hypoxia, epithelial-mesenchymal transition, angiogenesis, and immunity (Figure 8D and E). Overall, the high-risk group was significantly associated with pro-tumorigenic pathways. Notably, the lactylation-related pathway glycolysis was significantly and positively correlated with the risk score in the high-risk group (Figure 8D). We performed GSEA analysis of glycolysis in tumor and normal tissues. The results showed that glycolysis was significantly enriched in tumor tissues compared with normal tissues, suggesting a possible direct association with cervical carcinogenesis and progression (Figure 8F and G).

### Drug prediction and drug sensitivity analysis

**Figure 9:**
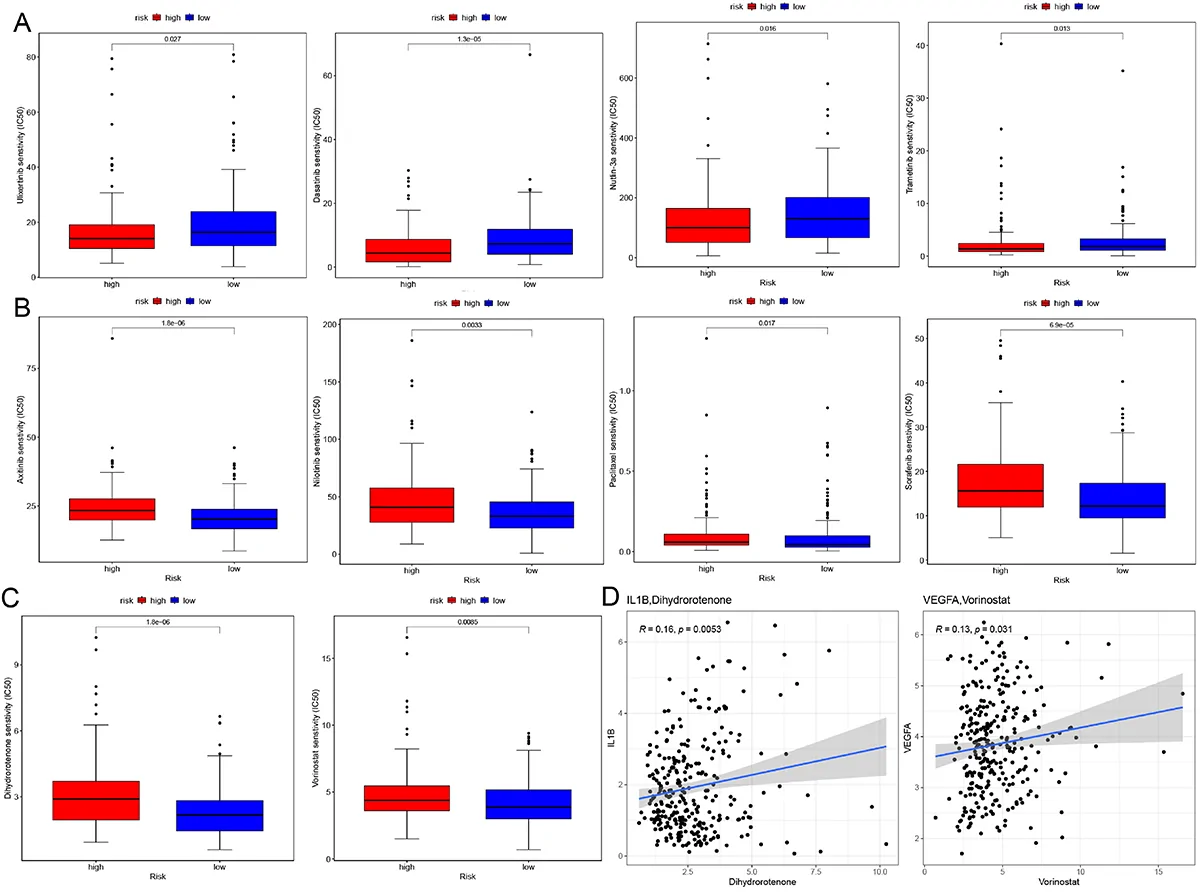
Drug prediction and drug sensitivity analysis. **A**, The chemotherapeutic drugs with lower maximal inhibitory concentrations (IC50) value in high-risk group. **B**, The chemotherapeutic drugs with lower IC50 value in low-risk group. **C**, Differences in IC50 values of dihydrorotenone or vorinostat in the high- and low-risk groups. Data are reported as median and interquartile range. Mann-Whitney test. **D**, Correlation between *IL1B* expression and IC50 values of dihydrorotenone (left). Correlation between *VEGFA* expression and IC50 values of vorinostat (right).

We calculated IC50 values for common drugs in the high- and low-risk groups. The results showed that the IC50s of ulixertinib, dasatinib, nutlin-3a, and trametinib were lower in the high-risk group (Figure 9A). IC50s of axitinib, nilotinib, paclitaxel, and sorafenib were lower in the low-risk group (Figure 9B). Correlation analysis showed that *ITGA5* was the only risk gene significantly associated with the above eight drugs (Supplementary Table S2). Furthermore, *ITGA5* and dasatinib had the highest correlation (R=-0.45), followed by *IL1B* and dasatinib (R=-0.4), suggesting that elevated expressions of *ITGA5* and *IL1B* were associated with increased sensitivity to dasatinib in CC patients.

Additionally, the DSigDB database was used to locate small molecule drugs for the risk genes. The results showed that dihydrorotenone targeted to *IL1B* (P*=*0.0167), vorinostat targeted to *VEGFA* (P*=*0.0043), and low-risk group patients were more sensitive to dihydrorotenone and vorinostat (Figure 9C). *IL1B* expression was positively correlated with the IC50 value of dihydrorotenone (R=0.16), and *VEGFA* expression was positively correlated with the IC50 value of vorinostat (R=0.13) (Figure 9D). Similarly, these results suggested that decreased expression of *IL1B* was associated with enhanced sensitivity to dihydrorotenone in CC patients, and decreased *VEGFA* was associated with enhanced sensitivity to vorinostat in CC patients.

### MR analysis

**Figure 10:**
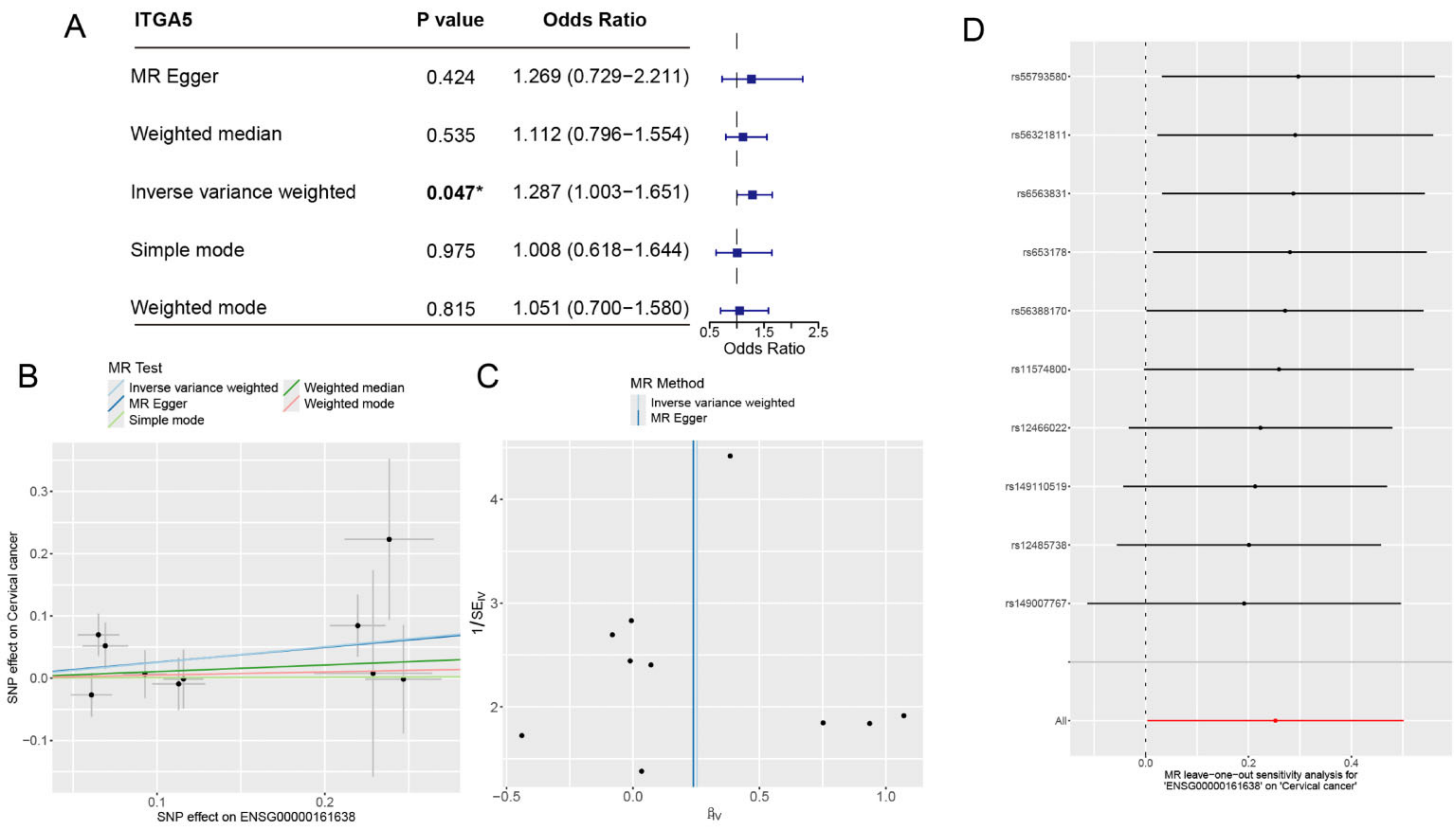
Mendelian randomization (MR) analysis of *ITGA5* and the risk of cervical cancer (CC). **A**, Forest plot based on five MR methods. **B**, Scatter plot. **C**, Funnel plot. **D**, Leave-one-out plot.

We performed MR analysis to investigate the potential causal relationship between risk genes and CC susceptibility. Using the IVW method, only *ITGA5* was identified as potentially associated with an increased risk of CC (odds ratio [OR]=1.287, 95% confidence interval [CI]=1.003-1.651, P*=*0.047) (Figure 10A-C). No evidence of horizontal pleiotropy or heterogeneity was detected (Supplementary Table S3). The result of the sensitivity analysis is presented in Figure 10D. Considering the relatively relaxed instrumental variable selection threshold and the limited sample size of CC cases, these results should be interpreted with caution and require further validation in larger studies.

### Risk gene expression validation

**Figure 11:**
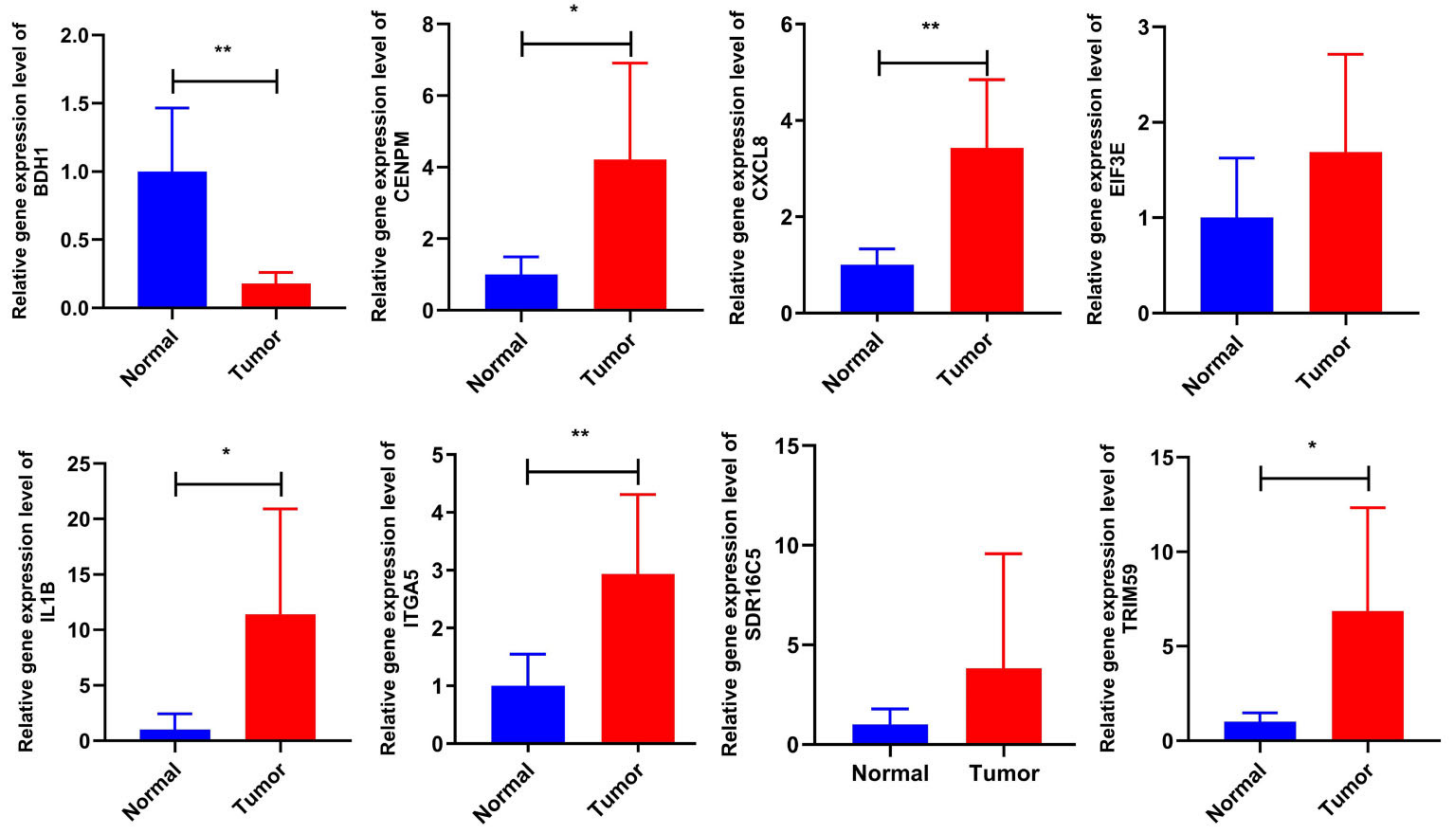
Validation of risk gene expression. Real-time qPCR was used to verify the expression of several risk genes between normal and tumor tissues from CC patients. Data are reported as median and interquartile range. *P<0.05, **P<0.01. Mann-Whitney test.

Eight genes of interest were selected for preliminary validation in tumor and adjacent normal tissues from six patients with CC (Figure 11). The results showed that *BDH1* was significantly downregulated in tumor tissues, whereas *CENPM*, *CXCL8*, *IL1B*, *ITGA5*, and *TRIM59* were significantly upregulated. In addition, *EIF3E* and *SDR16C5* exhibited an increasing trend in tumor tissues, although the differences did not reach statistical significance.

## Discussion

An increasing number of studies have highlighted the important role of lactylation in tumor biology. Recent evidence suggests that HPV can inhibit the lactylation of glucose-6-phosphate dehydrogenase at the K45 site, promoting the proliferation of CC cells by activating the pentose phosphate pathway (15). Furthermore, the upregulation of *DCBLD1* driven by lactylation could also influence the progression of CC (16). Therefore, targeted therapy against the lactylation pathway may play an important role in the treatment of CC.

scRNA-seq can detect differences in gene expression within individual cells and is useful for revealing heterogeneity within tumors. Using scRNA-seq data, we identified 630 genes associated with lactylation activity. Combined with bulk-seq data, we developed a risk model including 17 genes. Although these genes exhibit different expression patterns, they play critical roles in tumor progression, impacting the cell cycle (17) and multiple signaling pathways (18). Among them, some genes have been shown to be important in CC. For example, *CXCL8* (IL-8) has been associated with neutrophil recruitment and tumor cell proliferation in CC (19). Integrin alpha 5 (*ITGA5*) promotes endothelial tube formation in CC cells and is linked to poorer prognosis (20). Additionally, lactate secreted by cervical tumor cells could mediate the crosstalk between tumor cells and macrophages, leading to increased *IL1B* secretion (21). *BDH1*, which encodes a mitochondrial inner membrane dehydrogenase involved in cellular metabolic homeostasis, was downregulated in HeLa cells, consistent with our qPCR validation results (22). *TRIM59*, an E3 ubiquitin ligase, has been implicated in tumor progression and glycolytic regulation. Its functional role appears to be context-dependent across different cancer types, which may be related to the functional characteristics of the substrates (23,24). *TRIM59* has been reported to attenuate the pro-tumorigenic effects of M2-polarized tumor-associated macrophages in melanoma (25), indicating a potential immunomodulatory role. Taken together, these model genes play important roles in tumor prognosis, progression, and the immune microenvironment, and their specific functions in CC, as well as their potential associations with lactylation, warrant further investigation. Additionally, our risk model could accurately and independently predict the prognosis of CC patients and was related to clinical staging. The nomogram combining risk scores and clinical variables outperformed other clinical parameters, providing valuable guidance for clinical practice.

Lactate-mediated lactylation plays a significant role in the formation of the TME, promoting tumor progression by creating an immunosuppressive environment (26). In our study, functional enrichment analysis showed that the LRG risk score was associated with immunomodulatory pathways. Specifically, the risk score was negatively correlated with the infiltration levels of B cells and CD8^+^ T cells, and positively correlated with the infiltration levels of neutrophils. Previous studies suggest that neutrophils may facilitate immune escape mechanisms by suppressing T cell activity, thereby exerting an immunosuppressive effect in CC cells (27). Additionally, lactylation at the H3K18 site has been associated with the recruitment of neutrophils in the TME of liver metastases from colorectal cancer (28). Histone acetylation affects CD8 T cell metabolism and promotes CD8^+^ T cell dysfunction, which can lead to immune escape by malignant cells (29). In a highly glycolytic TME, lactate uptake by regulatory T cells enhances their inhibitory function and increases PD-1 expression, resulting in the failure of PD-1 blockade therapies (30). *CD274* (PD-L1) and *PDCD1LG2* (PD-L2) are known immune checkpoint molecules that interact with PD-1 and promote tumor immune escape (31). Our results suggest that *CD274* and *PDCD1LG2* were significantly upregulated in the high-risk group, which may help explain why this group exhibited higher TIDE scores.

Although TIDE was primarily developed and validated in melanoma and lung cancer cohorts, its application in other tumor types, including cervical cancer, should be regarded as providing a preliminary reference. Notably, previous studies have reported a close association between HPV positivity, PD-L1 expression, and CD8^+^ T-cell infiltration in CC (32,33). The consistency between elevated TIDE scores and the immunosuppressive features observed in our high-risk group suggested that TIDE may partially capture an immune-evasive phenotype in this context. Overall, the high-risk group associated with LRGs may experience immunosuppression and immune escape, contributing to a poor prognosis.

Alterations in genes are often linked to diseases. Lactylation enhances homologous recombination repair, which may affect DNA damage repair and thus the frequency of mutations in tumors (34). *TTN* and *PIK3CA* are common mutations associated with the pathogenesis and prognosis of CC (35). Our results showed a difference in the frequency of *TTN* and *PIK3CA* mutations between the high- and low-risk groups, which is crucial to understanding the roles of lactylation in disease progression and therapy. Previous studies indicate that lactylation is linked to tumor drug resistance, involving proteasome inhibitors (36), cisplatin (37), and monoclonal antibodies (38). To provide insights for clinical drug development, we evaluated drug sensitivity among patients in high- and low-risk groups, and ten drugs were identified by computational predictions. Among them, dasatinib has been reported to prevent CC cell invasion by inhibiting Src phosphorylation (39). Combination therapy with trametinib has been shown to reduce lactate production within the TME and lactylation at H3K18 in tumor-associated macrophages (40). Our risk model might help stratify patients for drug response, and it would be valuable to explore these predicted agents in future preclinical and clinical studies for personalized CC treatment.

This study employed a multi-dimensional approach to uncover differences in LRGs in CC with respect to clinical features, somatic mutations, immune cell infiltration, and drug sensitivity. These findings contribute to a deeper understanding of the biological mechanisms linking disease with lactylation, an emerging post-translational modification. Despite these strengths, our study has several limitations. First, the findings are based on bioinformatic analyses. Future research should focus on larger clinical cohorts and functional studies to fully elucidate the complex interactions among risk genes, lactylation patterns, and CC progression. This is important to translate these findings into effective clinical interventions. Second, the scRNA-seq data were only from a single individual, so these findings should be interpreted as exploratory. Third, although LASSO Cox regression was employed to reduce model complexity and perform feature selection, penalized regression approaches can be sensitive to data perturbations, particularly in high-dimensional settings and in the presence of correlated predictors. This inherent characteristic may lead to potential instability in feature selection. Fourth, the predictive performance of the model in the validation set was lower than that observed in the training set. This discrepancy, which is commonly encountered in prognostic modeling studies, suggests a potential degree of overfitting. Such performance differences may be attributable to limited sample size, high feature dimensionality, and the sensitivity of LASSO-based models to variations in the training data. Nevertheless, the model retained a certain level of discriminative ability in the external validation cohort, indicating preliminary generalizability. Future studies incorporating larger sample sizes, multicenter cohorts, or more robust resampling strategies may further enhance model stability and generalizability. Finally, the LASSO-Cox model used for risk prediction does not consider the biological functions of individual genes, which may limit its predictive performance across diverse datasets.

## Conclusion

In conclusion, our study developed a risk score model based on 17 LRGs. The model demonstrated independent prognostic value for clinical outcomes and may serve as a potential tool for risk stratification in CC. Furthermore, the high-risk group was associated with pro-tumorigenic pathways and immunosuppression, which may collectively contribute to a poorer prognosis. These findings were primarily based on bioinformatics analyses and provide a directional framework for subsequent experimental studies aimed at elucidating the underlying molecular mechanisms. The mechanistic roles and prognostic performance of these markers in cervical cancer require further validation in preclinical models and prospective clinical studies.

## Data Availability

The datasets used and/or analyzed during the current study are available from the corresponding author on reasonable request.

## Supplementary Materials

Click to view [pdf].
