# Supplementary material for: Bulk and single cell RNA sequencing data reveal lactylation-related gene signatures for prognosis and immunity in cervical cancer
Source: Braz J Med Biol Res. 2026 Aug 21;59:e15368. doi: 10.1590/1414-431X2026e15368 (PMC13502778; doi:10.1590/1414-431X2026e15368)
Supplement: Supplementary file 1 [file 1414-431X-bjmbr-59-e15368-suppl.pdf]

Figure S1. Clustering of malignant and normal epithelial cells based on single-cell sequencing data. **A**, Copy number variations (CNV) mapping shows heterogeneity of inferred CNVs between reference cells (fibroblasts) and tumor/epithelial cells. **B**, Heatmap displaying modified expression levels of genes across different samples, clustered using unsupervised clustering (K-means). **C**, Violin plot showing the distribution of CNV scores in the three K-means clusters. Clusters with higher CNV scores (clusters 1 and 3) are considered malignant cells. **D**, t-SNE plot displaying clusters of malignant and normal epithelial cells and **E**, the corresponding area under the curve (AUC) score distribution.

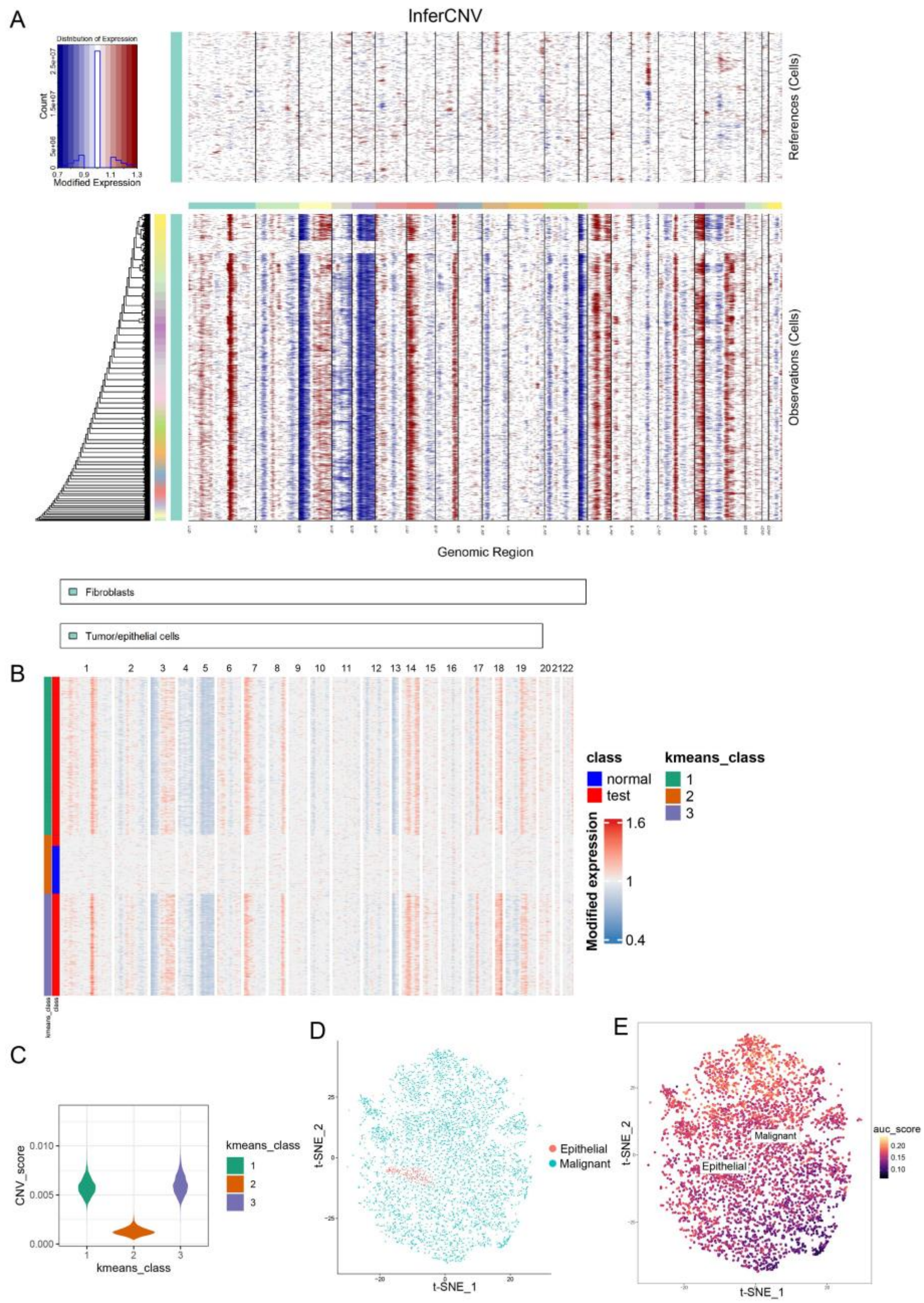

Figure S2. Differences in the expression levels of risk genes across different cell types.

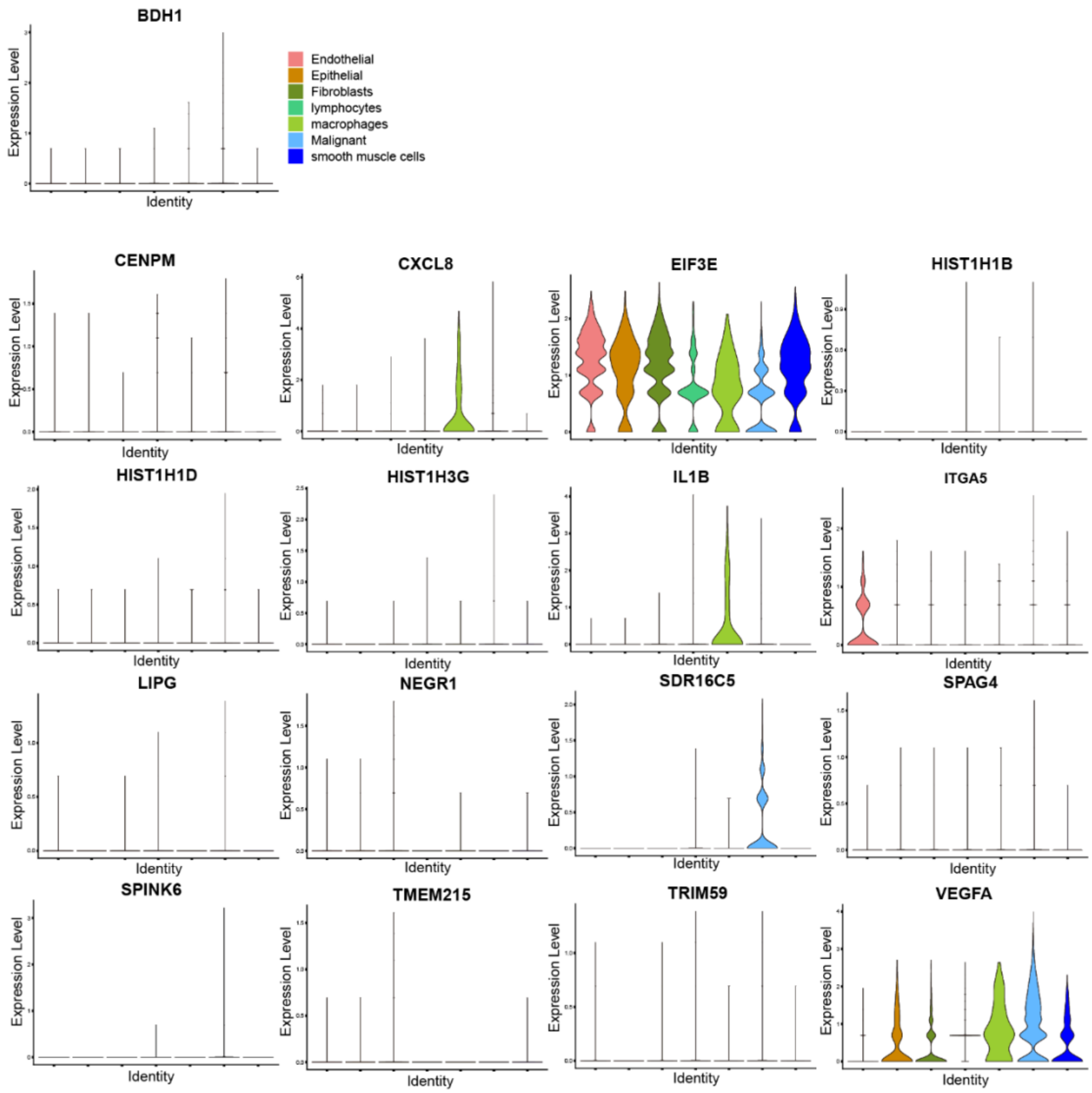

Figure S3. Lactylation-related genes (LRG) risk model for cervical cancer (CC) prognosis in the CGCI-HTMCP-CC cohort. **A**, Scatter plot of risk scores and survival status, and heatmap of the expression of 17 risk genes. **B**, Kaplan-Meier survival curves for overall survival of patients with high- and low-risk scores. **C**, ROC curves for 1- and 2-year predictions. **D**, Principal component analysis plot of patient samples in high- and low-risk groups.

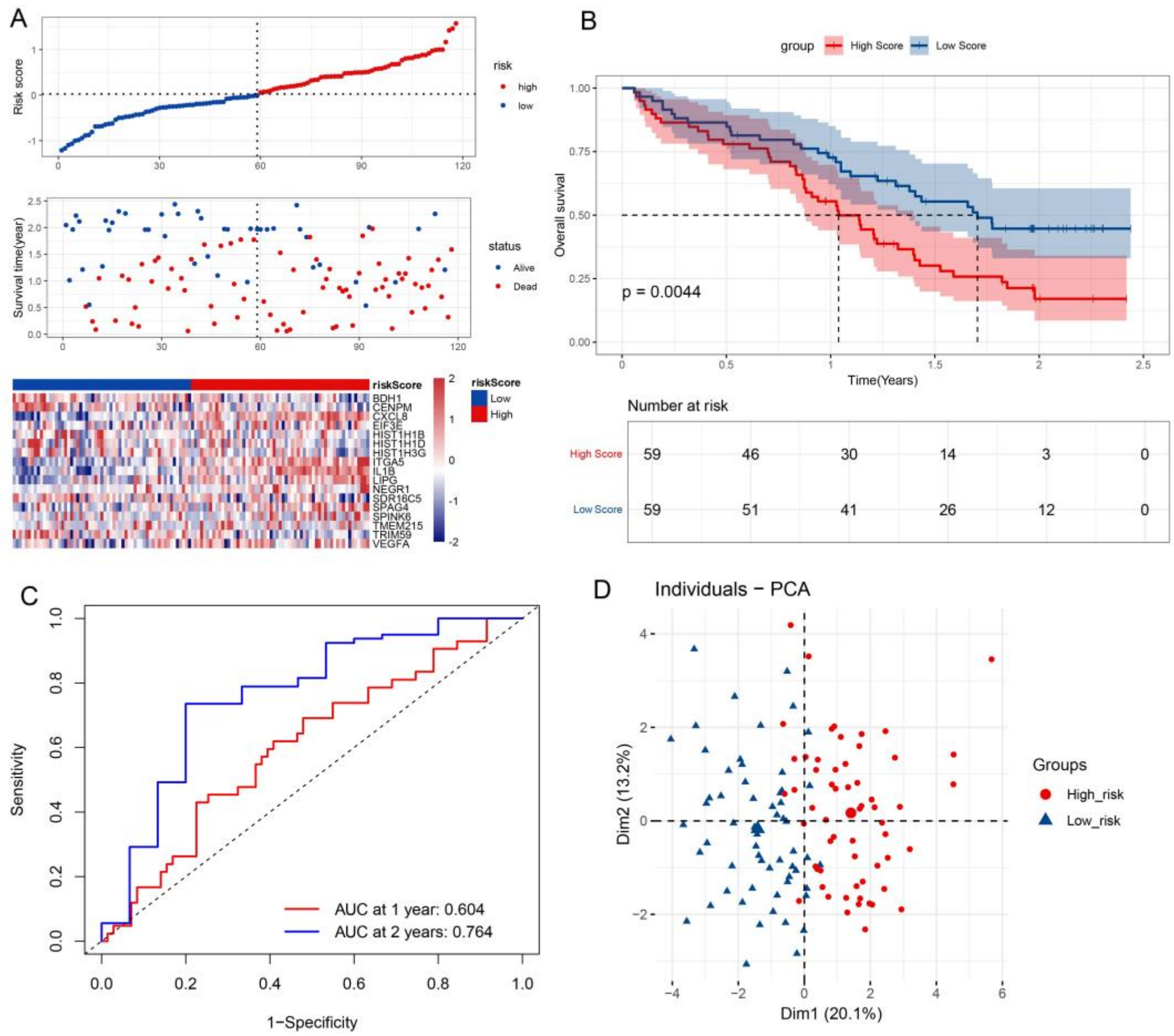

Figure S4. Lactylation-related genes (LRG) risk score-related nomogram for cervical cancer (CC) prognosis in the CGCI-HTMCP-CC cohort. **A**, ROC curves comparing the risk score, N staging, and nomogram for predicting 1-year survival in the TCGA cohort. **B**, ROC curves comparing the risk score, N staging, and nomogram for predicting 3-year survival in the TCGA cohort. **C**, Nomograms that include risk score and N staging could predict 1- and 2-year survival in the CGCI-HTMCP-CC cohort. **D**, ROC curves comparing the risk score, N staging, and nomogram for predicting 1-year survival in the CGCI-HTMCP-CC cohort. **E**, ROC curves comparing the risk score, N staging, and nomogram for predicting 2-year survival in the CGCI-HTMCP-CC cohort. **F**, Calibration curves for predicting 1- and 2-year survival.

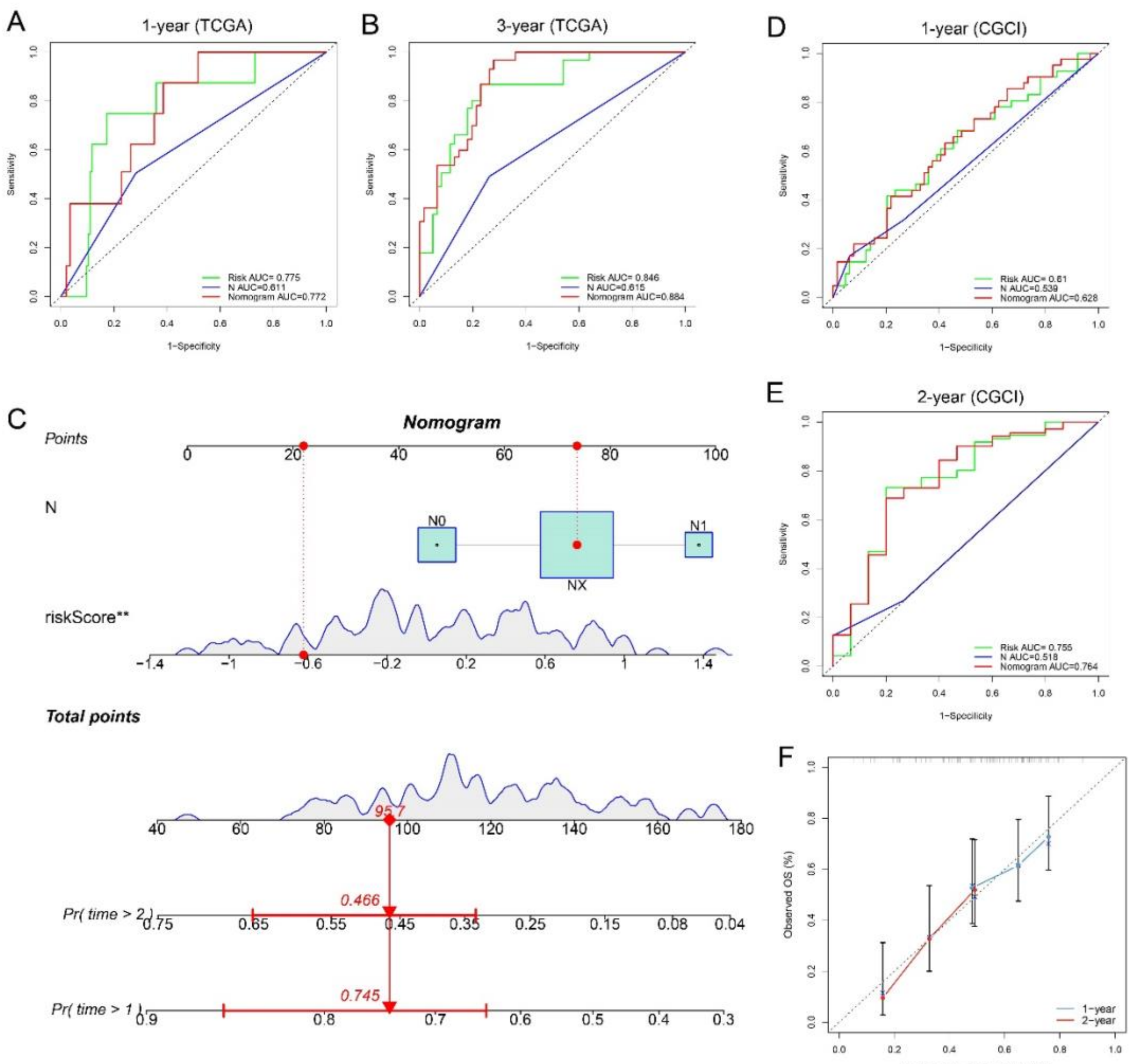

Table S1. List of primers for risk genes.

Gene name	Primer sequence (5' to 3')
GAPDH-F (reference gene)	GGAGCGAGATCCCTCCAAAAT
GAPDH-R (reference gene)	GGCTGTTGTCATACTTCTCATGG
ACTB-F (reference gene)	CATGTACGTTGCTATCCAGGC
ACTB-R (reference gene)	CTCCTTAATGTCACGCACGAT
ITGA5-F	CATGCACTGTGAAGGACCCT
ITGA5-R	AGTCCTTGTGGCATGTCTGG
CXCL8-F	GCAGAGGGTTGTGGAGAAGT
CXCL8-R	TGGCAACCCTACAACAGACC
IL1B-F	GAAGTACCTGAGCTCGCCAG
IL1B-R	CATGGCCACAACAACTGACG
CENPM-F	CCAGAACACAGAGGAGTCCC
CENPM-R	CAGGGACAGCAGGTTCAGAG
TRIM59-F	AAGATCCTCGTGTACTGCCAT
TRIM59-R	CAATGCCAGTTGGAGCAATTTC
EIF3E-F	TGCAATTCAGACAATGTGTCCA
EIF3E-R	TTAGAACCTGCCGACGTTTTC
SDR16C5-F	TCCAGAGGACACACAGCTCT
SDR16C5-R	CACTCGGTCTGCGTATCCAG
BDH1-F	ACACACACACCCCTGTTCTG
BDH1-R	TTCGGTCCCAACATACAGGC

Table S2. Correlation analysis between risk genes and drug.

	Ulixertinib	Dasatinib	Nutlin-3a	Trametinib	Axitinib	Nilotinib	Paclitaxel	Sorafenib
BDH1	R=–0.29<br>P=5e-07	R=0.25<br>P=1.1e-05	ns	ns	R=–0.27<br>P=2.5e-06	R=–0.15<br>P=0.012	ns	R=–0.25<br>P=1.9e-05
CENPM	R=0.14<br>P=0.014	R=0.19<br>P=0.0013	R=0.16<br>P=0.0078	ns	R=–0.36<br>P=3.6e-10	R=–0.26<br>P=5.5e-06	ns	R=–0.28<br>P=1e-06
CXCL8	R=–0.12<br>P=0.045	R=–0.14<br>P=0.021	ns	ns	R=0.24<br>P=4e-05	R=0.19<br>P=0.0011	ns	R=0.28<br>P=1.2e-06
EIF3E	R=–0.26<br>P=9.6e-06	R=–0.15<br>P=0.0094	ns	ns	ns	R=–0.3<br>P=1.5e-07	R=–0.15<br>P=0.0093	R=–0.23<br>P=7.2e-05
HIST1H1B	R=–0.14<br>P=0.017	ns	ns	ns	R=–0.17<br>P=0.0044	R=–0.24<br>P=4.8e-05	R=–0.15<br>P=0.011	R=–0.16<br>P=0.0075
HIST1H1D	ns	ns	ns	ns	ns	R=–0.17<br>P=0.0046	ns	ns
HIST1H3G	ns	ns	ns	ns	ns	R=–0.13<br>P=0.031	ns	ns
IL1B	R=–0.25<br>P=1.8e-05	R=–0.4<br>P=8.6e-13	R=–0.13<br>P=0.024	R=–0.23<br>P=5.4e-05	R=0.24<br>P=4.3e-05	ns	R=0.19<br>P=0.00097	ns
ITGA5	R=–0.22<br>P=0.00016	R=–0.45<br>P=7.1e-16	R=–0.15<br>P=0.011	R=–0.2<br>P=0.00019	R=0.28<br>P=1.2e-06	R=–0.21<br>P=0.00034	R=–0.14<br>P=0.021	R=0.3<br>P=2e-07
LIPG	ns	R=–0.17<br>P=0.0035	ns	ns	R=0.17<br>P=0.0032	R=0.24<br>P=2.8e-05	ns	R=0.31<br>P=9.4e-08
NEGR1	ns	ns	ns	ns	R=0.13<br>P=0.029	R=0.13<br>P=0.032	ns	ns
SDR16C5	R=–0.29<br>P=3.9e-07	R=–0.13<br>P=0.029	ns	R=–0.31<br>P=6.2e-08	R=–0.14<br>P=0.021	ns	R=–0.19<br>P=0.00093	ns
SPAG4	R=0.17<br>P=0.0033	R=0.13<br>P=0.028	ns	R=0.16<br>P=0.0071	ns	R=0.19<br>P=0.0015	R=0.31<br>P=8.7e-08	R=0.2<br>P=0.00067
SPINK6	R=0.2<br>P=0.00054	R=–0.18<br>P=0.0023	ns	R=–0.14<br>P=0.017	R=0.25<br>P=1.1e-05	R=0.13<br>P=0.028	ns	ns
TMEM215	ns	ns	ns	ns	ns	R=0.17<br>P=0.0039	R=0.16<br>P=0.0063	R=0.17<br>P=0.004
TRIM59	ns	ns	ns	ns	R=–0.13<br>P=0.031	R=–0.14<br>P=0.015	R=–0.17<br>P=0.0044	ns
VEGFA	ns	ns	ns	ns	ns	ns	ns	R=0.17<br>P=0.0031

ns: not statistically significant.

Table S3. Horizontal pleiotropy and heterogeneity tests for Mendelian randomization (MR) analysis.

Outcome	Exposure	Horizontal pleiotropy test			
			Egger_intercept	se	pvalue
Cervical cancer	ENSG00000161638 (ITGA5)		0.0017941	0.0325015	0.957332
		Heterogeneity test			
		Method	Q	Q_df	Q_pval
		MR Egger	8.6802245	8	0.3699856
		Inverse variance weighted	8.6835307	9	0.4669849
